# Approximation method for yielding support analysis in high ground stress soft surrounding rock tunnels

**DOI:** 10.1371/journal.pone.0299426

**Published:** 2024-03-13

**Authors:** Zhiming Xu, Biao Dong, Yashuang Bai

**Affiliations:** Institute of Civil Engineering, Southwest Forestry University, Kunming, China; University of Vigo, SPAIN

## Abstract

In solving the whole process of interaction between soft rock and yielding support in high-stress environments in tunnels using mechanical analysis methods, it is challenging to simultaneously satisfy both displacement coordination and static equilibrium at the contact surface between the rock and the support structure. This paper, based on the mechanical analysis of rock and rigid support, considers the impact of the circumferential installation of yielding elements on radial displacement, and proposes displacement approximation and support force approximation methods using displacement coordination and static equilibrium as approximation conditions. The study fits curves of numerical simulation results and laboratory test results of yielding elements, and attempts to directly use the laboratory test data set of yielding elements as computational data. By calculating two circular tunnel examples and comparing the effects of the trisection method, bisection method, and substitution method on the convergence of the displacement approximation method, the effectiveness of the methods proposed in this paper is verified. The research results show that the two approximation algorithms proposed in this paper have good accuracy and reliability in calculating the relative displacement of rock and yielding support structure contact surfaces, and the support force of yielding support. The bisection method outperforms the trisection and substitution methods in terms of stability and convergence. However, there are certain limitations in this study, such as the effectiveness of the algorithm may be influenced by geological conditions; the complexity of actual geological conditions may exceed the assumptions of the current rock-support mechanical analysis model.

## 1 Introduction

Using yielding support instead of rigid support is an effective method to solve the problem of large deformation in soft rock tunnels under high ground stress conditions [1]. Engineering practice has shown that increasing the stiffness of support to resist large deformations of soft rock by means of strong support and hard top often fails to meet the support needs and frequently requires multiple replacements or reinforcement of the support structure [2]. This not only increases the project budget and extends the construction period but also puts the support structure under high stress, increasing the risk of failure[3]. Yielding support is an improvement based on rigid support, such as installing yielding elements around the circumference of the initial rigid support[4]. Yielding support allows for deformation of the support to release the energy of the surrounding rock and reduce the pressure of the surrounding rock, thereby improving the safety of the support [5], and is widely used in tunnels with high ground stress and weak surrounding rock. With the widespread application of yielding support in tunnels with high ground stress and weak surrounding rock, it is of great engineering significance to accurately consider the interaction mechanism between the surrounding rock and the yielding support system.

To reveal the interaction mechanism between the surrounding rock and the yielding support, scholars at home and abroad have conducted in-depth studies on this mechanism using methods such as numerical simulation and mechanical analysis. For example, numerical simulation methods have been used by researchers like Lian Chuanjie [6–8] to analyze the design parameters, simulation methods, and support effects of prestressed yielding anchor bolts; Wang Bo and others [9] constructed a yielding support system consisting of yielding anchor bolts, compressible steel arches, and deformation slots or yielding controllers to study the effects of the initial yielding point and yielding amount on the release of strain energy of the surrounding rock and support bearing; Tian and others [10] established a combined yielding support system of U-shaped steel frames and compressible foam concrete, analyzing the relationship between the thickness of the compressible layer and large deformations of soft rock; Qi Chun and others [11] used the Mohr-Coulomb criterion to establish a beam-spring model to simulate the contact effect between segmental lining and compressible layers, and a stiffness reduction model for compressible layers to analyze the effect of the presence or absence of compressible layers, their stiffness, and thickness on the internal force and deformation distribution of the support; Liu Fang and others [12] simulated the surrounding rock with the Cvisc creep model, using particle elements, solid elements, cable elements, and beam elements to simulate gravel filling layers, surrounding rock, grouting layers, anchor bolts, and linings, establishing a combined support model of segmental lining-compressible layer-anchor bolts. Hu and others [13] compared the yielding performance of two compressible materials (compressible concrete and expandable clay), illustrating the interaction mechanism between segmental lining and yield materials. However, the calculation accuracy of the numerical simulation method is highly related to the modeling quality of the yielding elements, susceptible to human factors, and the universality and predictive ability of numerical models need to be improved.

In the analysis of support displacement, yielding amount is added for calculation. Lei Sheng xiang and others [14] used the structural mechanics method to analyze the deformation pattern of the support structure and gave suggestions on the installation position of the circumferential yielding elements; Tian Yun and others [15] used the circular tunnel perimeter elastoplastic displacement formula, considering the aging weakening characteristics of the surrounding rock and the resistance of the buffer layer support, to propose a buffer layer parameter design method; Wu Kui and others [16–19] used an improved fractional order Burgers creep model to derive an analytical solution for tunnel displacement and support pressure considering the effects of the tunnel face and delayed support action; Dong Jianhua and others [20] derived the full process analytical solution for elastoplastic deformation of the surrounding rock under the action of yielding support. Hu Xiongyu and others [21] established a theoretical model of the interaction between the surrounding rock, the compressible layer of expanded clay, and the segmental lining, and determined the calculation method for the thickness design parameters of the expanded clay compressible layer. Yang Kai and others [22] calculated the radial shrinkage caused by the circumferential yielding amount in circular tunnels, adding this radial shrinkage to the radial support displacement and then calculating the new support force and surrounding rock displacement. However, the displacement of the surrounding rock around the tunnel calculated in a single calculation by superimposing the yielding amount is not equal to the displacement of the top of the yielding support, and the result only satisfies the static equilibrium condition, not the displacement coordination condition.

Therefore, to address the limitations of existing methods, this paper proposes a new approximation method for solving. This study is based on the interaction mechanism between rock and support, focusing on the tunnel steel arch yielding elements proposed by Dong Biao [23] and others as the research subject. In this study, we attempt to directly use the laboratory test data set of yielding elements as computational data and apply numerical approximation algorithms to calculate the relative displacement at the contact surface between the rock and yielding support, as well as the support force of the support structure. The core of the numerical approximation algorithm proposed in this paper lies in accurately calculating the relative displacement at the contact surface between the rock and yielding support and the support force of the support structure, analyzing the entire interaction process between rock and yielding support.

Additionally, through comparative analysis of numerical simulation results and laboratory test results of yielding elements, combined with numerical approximation algorithms, this study highlights the necessity of using the proposed approximation method in solving yielding support issues. This method not only satisfies the static equilibrium conditions but also meets the displacement coordination conditions, providing a solution closer to practical engineering needs. Finally, by analyzing two circular tunnel examples, the feasibility of the numerical approximation algorithm in solving yielding problems is further verified.

## 2 The surrounding rock-support interaction model

**Fig 1:**
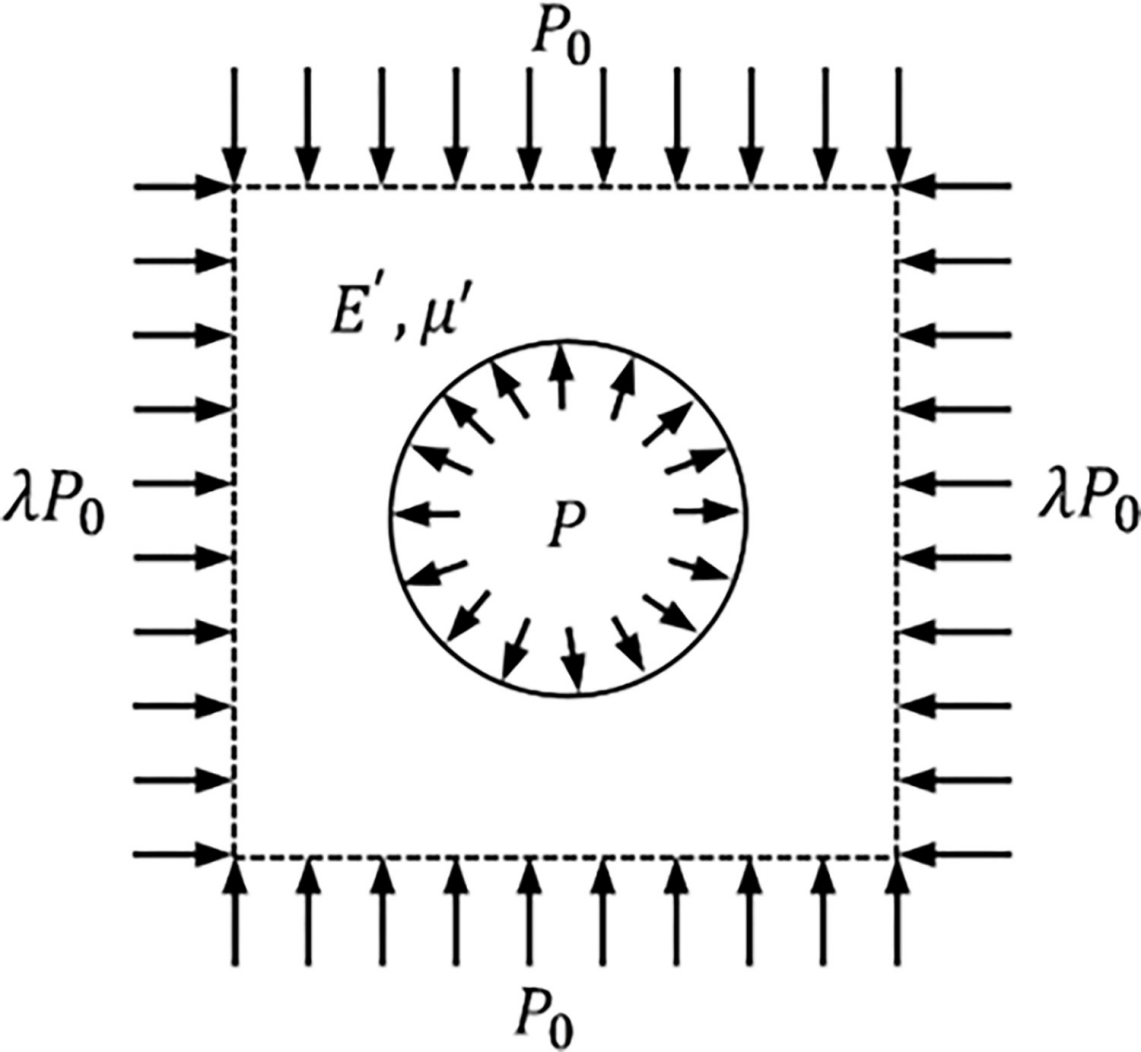
Tunnel excavation model.

**Fig 2:**
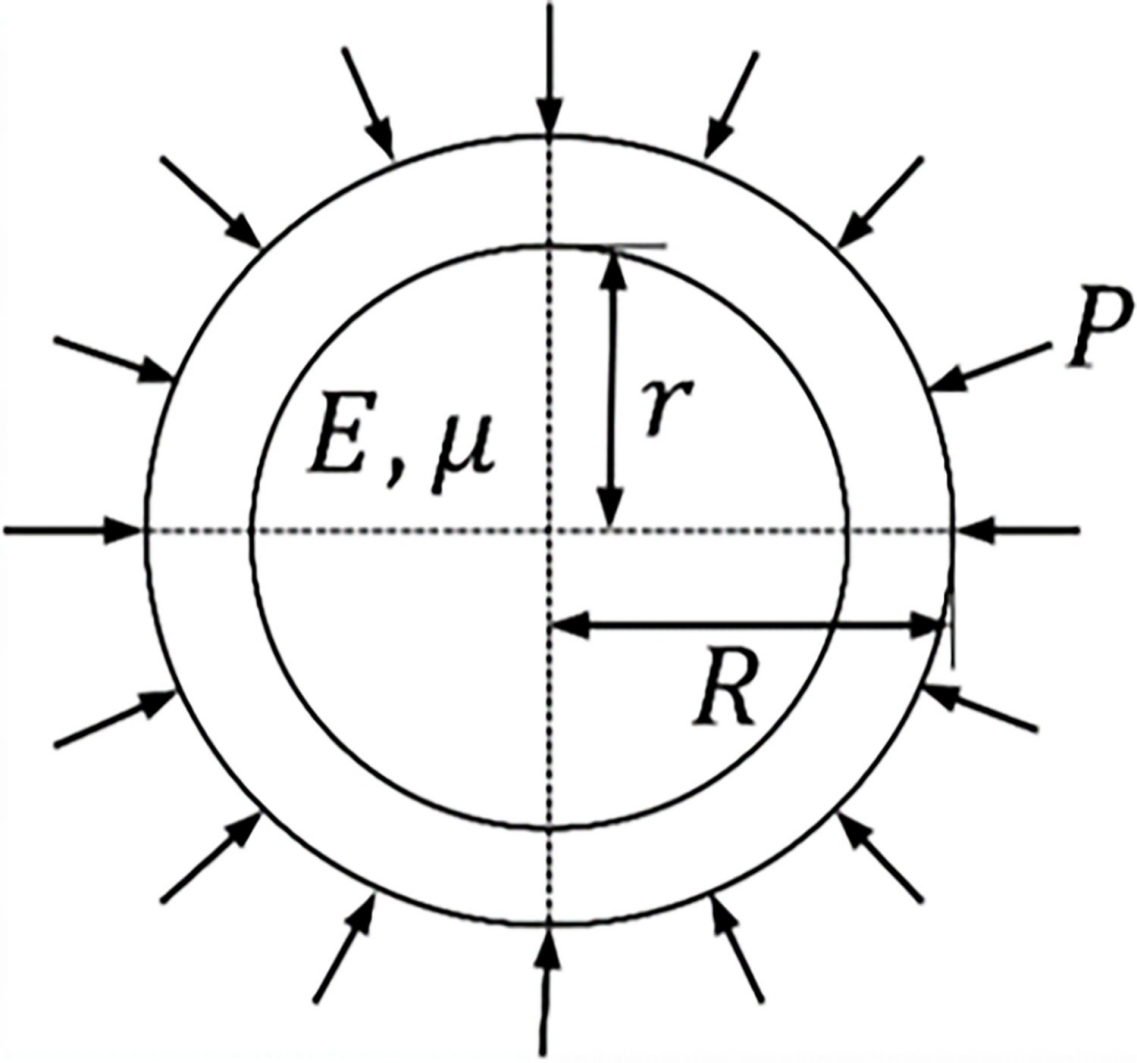
Rigid support mechanical model.

In analyzing the interaction mechanisms of elastic surrounding rock-rigid support and elastoplastic surrounding rock-rigid support, it is assumed that the initial ground stress of the rock mass is *P*_0_ and the support resistance is *P* The tunnel excavation model is shown in Fig 1, and the rigid support mechanical model is depicted in Fig 2.

### 2.1 Support elastic solution

The displacement and stress of rigid support under uniform pressure *P* are calculated using the Lamé solution, as follows:

$$\sigma_{\rho }=-\frac{1-\frac{r^{2}}{\rho^{2}}}{1-\frac{r^{2}}{R^{2}}}P$$ (1)

$$\sigma_{\varphi }=-\frac{1+\frac{r^{2}}{\rho^{2}}}{1-\frac{r^{2}}{R^{2}}}P$$ (2)

$$u_{\rho }=-\frac{\left(1+\mu \right)}{E(1-\frac{r^{2}}{R^{2}})}P[\frac{r^{2}}{\rho }+(1-2\mu )\rho ]$$ (3)

where *σ*_*ρ*_, *σ*_*φ*_, and *u*_*ρ*_ represent radial stress, circumferential stress, and radial displacement, respectively.

### 2.2 Elastic solution for surrounding rock

The relative displacement of the elastic surrounding rock [24,25] can be expressed as

$$u_{\rho }^{\prime }=-\frac{(1+\mu^{\prime })R^{2}}{E^{\prime }\rho }[-P+\frac{P_{0}}{2}\{(1+\lambda )+[4(1-\mu )-\frac{R^{2}}{\rho^{2}}](1-\lambda )cos2\varphi \}]$$ (4)

When *ρ* = *R*, the relative displacement around the rock tunnel is

$$u_{R}^{\prime }=-\frac{R(1+\mu^{\prime })}{E^{\prime }}\{-P+\frac{P_{0}}{2}[(1+\lambda )+(3-4\mu^{\prime })(1-\lambda )cos2\varphi ]\}$$ (5)

The radial displacement of the support’s top is

$$u_{R}=-\frac{\left(1+\mu \right)}{E(1-\frac{r^{2}}{R^{2}})}P[\frac{r^{2}}{R}+(1-2\mu )R]=-\frac{R(1+\mu )}{E}\frac{\left(r^{2}+(1-2\mu )R^{2}\right)}{R^{2}-r^{2}}$$ (6)

When the displacement coordination condition is satisfied at the contact surface between the surrounding rock and the support, $u_{R}=u_{R}^{\prime }$ the support force can be calculated using Eqs (5) and (6)

$$P=\frac{\frac{P_{0}}{2}[(1+\lambda )+(3-4\mu^{\prime })(1-\lambda )\cos2\varphi ]}{\frac{E^{\prime }(1+\mu )}{E(1+\mu^{\prime })}\frac{\left\{r^{2}+(1-2\mu )R^{2}\right\}}{R^{2}-r^{2}}+1}$$ (7)

If the displacement $u_{R}^{\prime }$ around the rock tunnel is known, the support force can be calculated using Eq (5), which is

$$P=\frac{E^{\prime }u_{R}^{\prime }}{R(1+\mu^{\prime })}+\frac{P_{0}}{2}[(1+\lambda )+(3-4\mu^{\prime })(1-\lambda )cos2\varphi ]$$ (8)

### 2.3 Elastic solution for surrounding rock

When not considering the volume expansion equation of elastoplastic bodies, the formula for calculating the elastoplastic displacement of general circular tunnels [23] is:

$$u_{\rho }^{\prime }=\frac{1}{4G\rho }[R_{p}^{2}+(1+\lambda )R_{p}f(\varphi )]*\{sin\theta [(1+\lambda )P_{0}+2Ccot\theta ][1+\frac{(1-\lambda )sin\theta }{R_{p}(1-sin\theta )}f(\varphi )]-P_{0}(1-\lambda )\cos2\varphi \}$$

$$R_{p}=R{\left\{\frac{\left[(1+\lambda )P_{0}+2C\cot\theta ](1-\sin\theta \right\}}{P+2C\cot\theta }\right\}}^{\frac{1-\sin\theta }{2\sin\theta }}$$

$$f(\varphi )=\frac{2R_{p}(1-\sin\theta )P_{0}}{\left[(1-\lambda )P_{0}+2C\cot\theta \right]}$$ (9)

where *R*_*p*_ is the radius of the plastic zone, *G* is the shear modulus, *C* and *θ* are the cohesion and internal friction angle of the rock, and *φ* is the position angle.

### 2.4 Support yielding displacement

The displacement coordination relationship mentioned in this paper refers to the consistent relative radial displacement around the tunnel between the surrounding rock and the support. After installing yielding elements circumferentially on the support, it is necessary to convert the circumferential yielding displacement of the support into radial yielding displacement based on the geometric relationship inside the tunnel. Before determining the circumferential yielding displacement, the concentrated force on the yielding elements should be calculated first, then search the circumferential yielding displacement *u*_*i*_ corresponding to this concentrated force from the loading test data set. The formula for calculating the concentrated force is as follows:

$$F=\sigma_{\varphi }A$$ (10)

where A is the contact area between the yielding element and the rigid support.

Assuming that the circumferential yielding displacement is uniformly distributed along the midline of the circular ring, and the number of yielding elements is *n* the total radial yielding displacement *u*^*n*^ is calculated based on the geometric relationship as

$$u^{n}=\frac{R+r}{2R}\frac{1}{2\pi }\sum_{i}^{n}u_{i}$$ (11)

By adding the total radial yielding displacement *u*_*n*_ to the radial displacement *u*_*R*_ of the rigid support, the total radial displacement of the yielding support can be obtained $u_{R}^{*}$

$$u_{R}^{*}=u_{R}+u^{n}$$ (12)

## 3 The proximity algorithm

**Fig 3:**
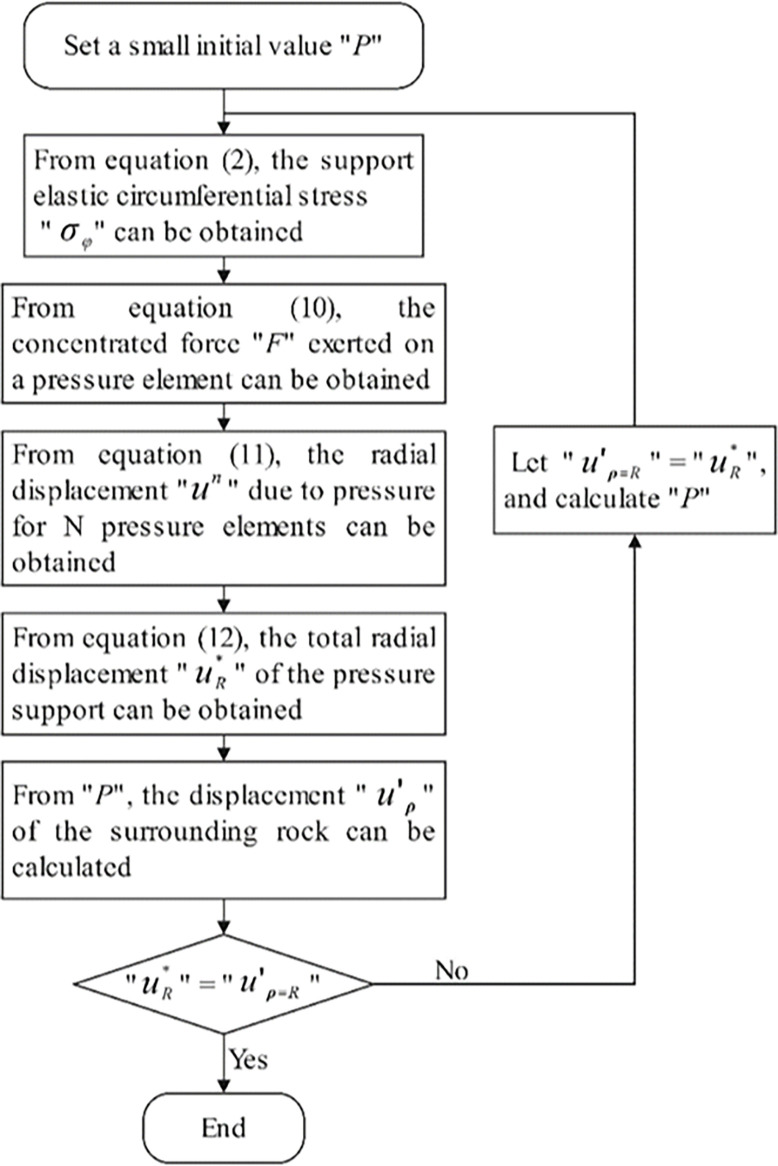
Numerical approximation algorithm technology roadmap.

Using Formula (10), the concentrated force on the pressure components is calculated from the rigid support ring’s stress. This force is then used to search for experimental data on pressure components to obtain the displacement due to pressure. However, this method of obtaining displacement due to pressure does not take into account the impact of pressure action on the circumferential stress of the support ring. Compared to the actual displacement due to pressure on the support, its value is larger. The relative displacement of the pressure support calculated using Formula (12) at the interface between the support and the surrounding rock will struggle to meet the displacement coordination conditions. Furthermore, if only the displacement coordination condition between the surrounding rock and the pressure support is considered, without considering the static equilibrium condition, the calculated displacement value and support force will be inaccurate. To address the difficulty of simultaneously meeting the displacement coordination and static equilibrium conditions at the contact surface, this paper proposes the displacement proximity method and the support force proximity method. The numerical approximation algorithm technology roadmap is shown in Fig 3.

### 3.1 The displacement proximity method

The displacement approximation method involves using the bisection method to approximate the displacement around the surrounding rock tunnel to the displacement of the support top plate. The displacement of the surrounding rock and the support force are calculated and updated until the displacement coordination condition (convergence) is met. The specific steps are as follows:

1) Calculate the support force *P* using Formula (7);

2) Let *ρ* = *R*, calculate the circumferential stress *σ*_*φ*,*R*_ and the radial displacement *u*_*R*_ of the support using Formulas (2) and (3);

3) Substitute *σ*_*φ*,*R*_ into Formula (10) to calculate the concentrated force *F*, and search for the corresponding displacement *u*_*i*_ from the displacement-load data of the pressure components;

4) Substitute *u*_*i*_ into Formulas (11) and (12) to calculate the total radial displacement of the support $u_{R}^{*}$;

5) Calculate the radial displacement around the rock tunnel $u_{R}^{\prime }$ using Formula (5);

6) Determine whether $u_{R}^{\prime }$ equals $u_{R}^{*}$

6.1) If yes, the solution is complete;

6.2) If not, set $u_{R}^{\prime }=(u_{R}^{\prime }+u_{R}^{*})/2$, calculate the support force *P* using Formula (8), and return to step 2) for further iteration.

### 3.2 Support force approximation method

For situations where it is inappropriate to pre-calculate the support force using the displacement coordination condition, this paper proposes a support force approximation algorithm. This algorithm requires an initial assumption of the support force range, suggested to be [0, 10*P*_0_]. Based on the positional relationship between the displacement around the rock tunnel and the radial displacement of the support, the support force range is gradually narrowed by comparing whether the displacement calculated with the preset support force meets the displacement coordination condition. The specific steps are as follows:

1) Assume *P*_down_ = 0, *P*_up_ = 10*P*_0_ and preset the support force *P* = (*P*_down_+*P*_up_)/2;

2) Let *ρ* = *R*, calculate the circumferential stress *σ*_*φ*,*R*_ and radial displacement *u*_*R*_ of the support using Formulas (2) and (3);

3) Substitute *σ*_*φ*,*R*_ into Formula (10) to calculate the concentrated force *F*, and search for the corresponding displacement *u*_*i*_ from the displacement-load data of the pressure components;

4) Substitute *u*_*i*_ into Formulas (11) and (12) to calculate the total radial displacement of the support $u_{R}^{*}$;

5) Calculate the radial displacement around the rock tunnel $u_{R}^{\prime }$ using Formula (5);

6) Determine whether $u_{R}^{\prime }$ equals $u_{R}^{*}$:

6.1) If yes ($u_{R}^{\prime }=u_{R}^{*}$), the solution is complete;

6.2) If not, compare the sizes of $u_{R}^{\prime }$ and $u_{R}^{*}$, update parameters, and return to step 1) for further iteration;

6.2.1) If $u_{R}^{\prime }>u_{R}^{*}$, then *P*_*start*_ = *P*;

6.2.2) If $u_{R}^{\prime }<u_{R}^{*}$, then *P*_*end*_ = *P*;

## 4 Construction of yielding elements

**Fig 4:**
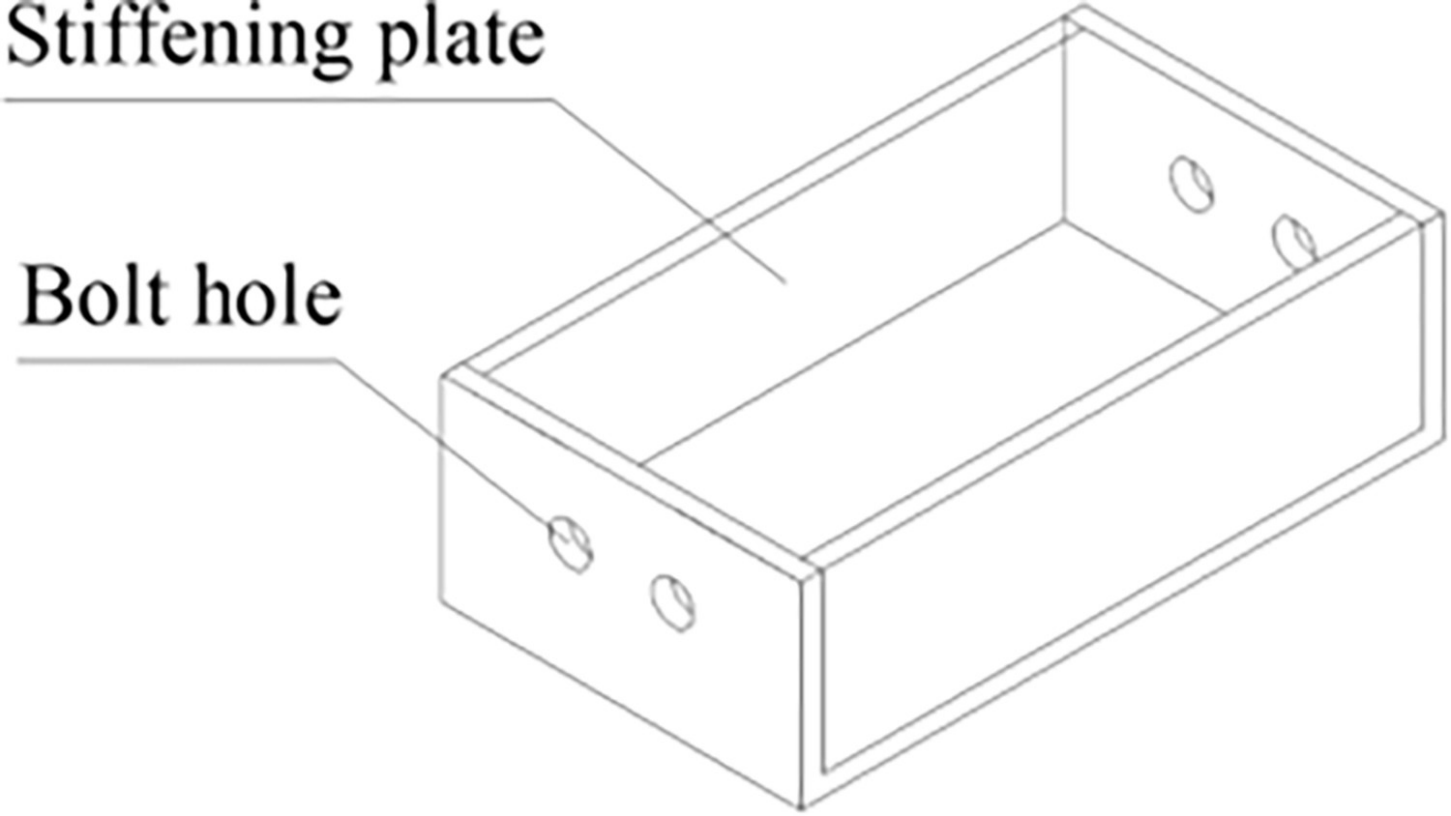
Inner flange steel.

**Fig 5:**
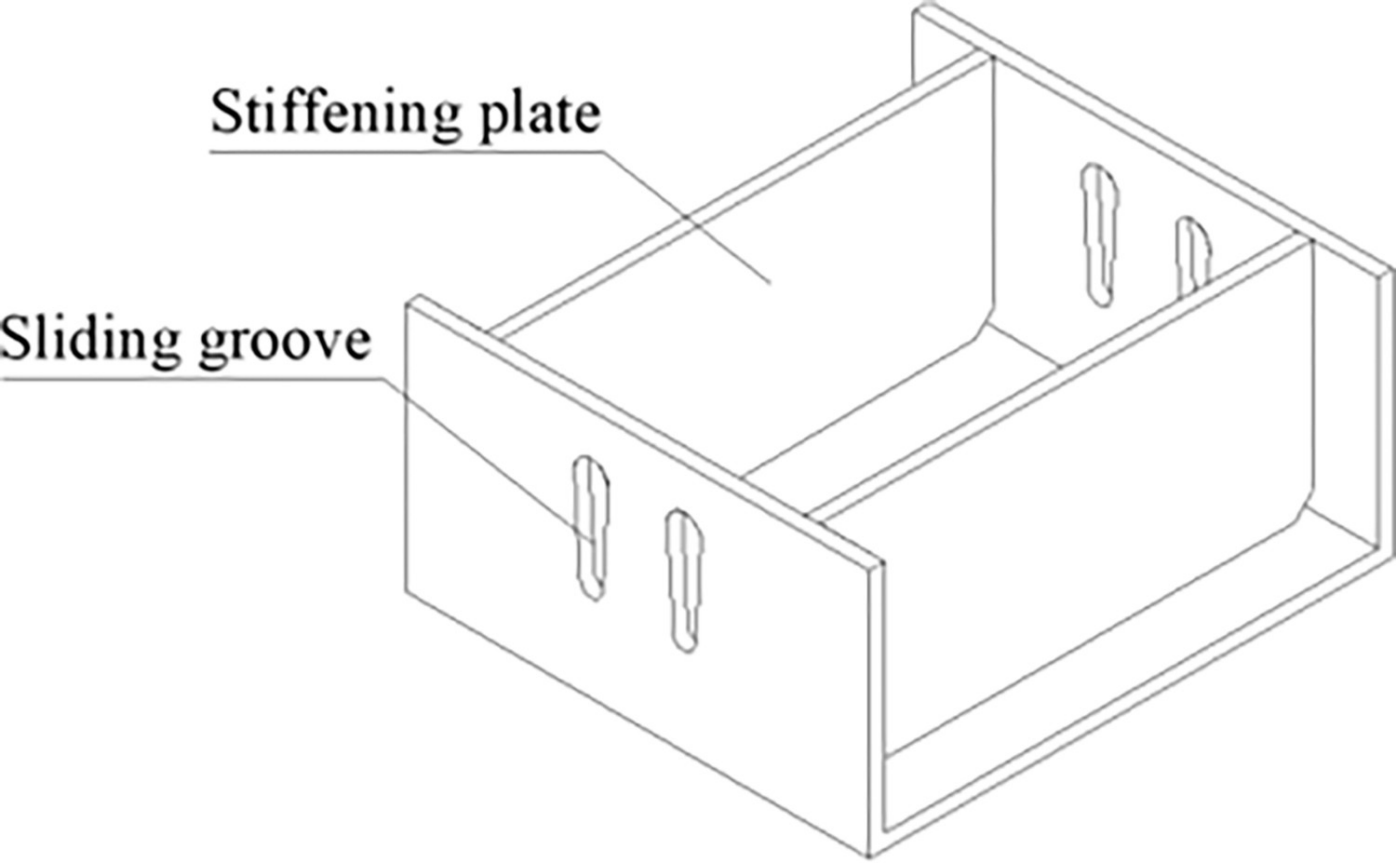
Outer flange steel.

**Fig 6:**
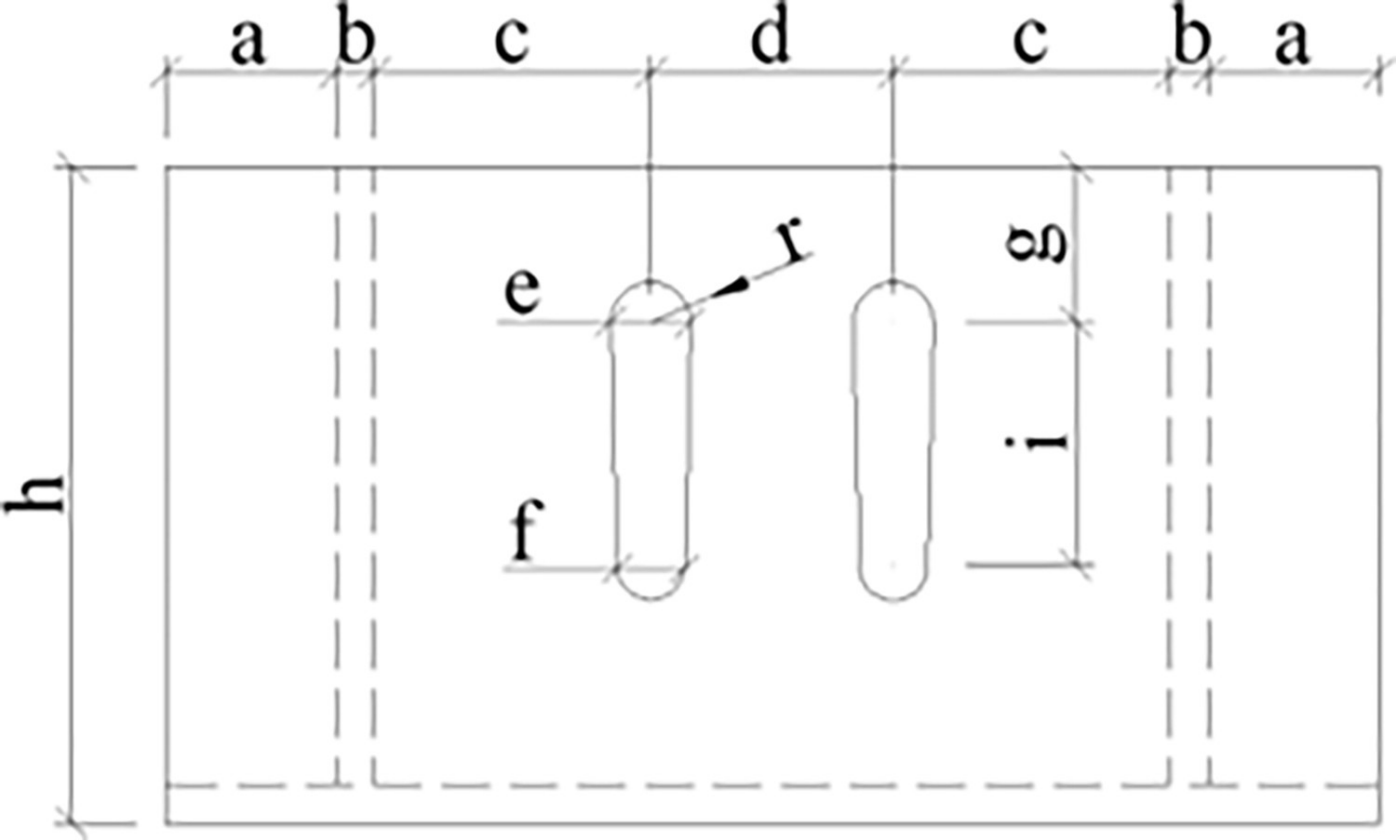
Dimensions of groove surface.

The yielding element is composed of an inner channel steel, an outer channel steel, and high-strength bolts, where the top of the inner channel steel and the bottom of the outer channel steel are connected to Q235 I-beam steel, respectively. The inner and outer channel steels are connected by GT/B 1228M16×60 high-strength bolts. The cross-sections of the inner channel steel, outer channel steel, and slide channel are shown in Figs 4–6, respectively, with detailed parameters of the channel steel and slide channel surface presented in Table 1 [23].

**Table 1:** Detailed parameters of the channel steel and the groove surface.

Channel steel size Unit: mm										
Component					Length		Width		Height	
Inner flange steel					250		140		77	
Outer flange steel					280		250		135	
Dimensions of groove surface Unit: mm										
a	b	c	d	e		f	g	h	i	r
35	8	57	50	17		15	32	127	50	8.5

## 5 Numerical simulation of yielding elements

**Fig 7:**
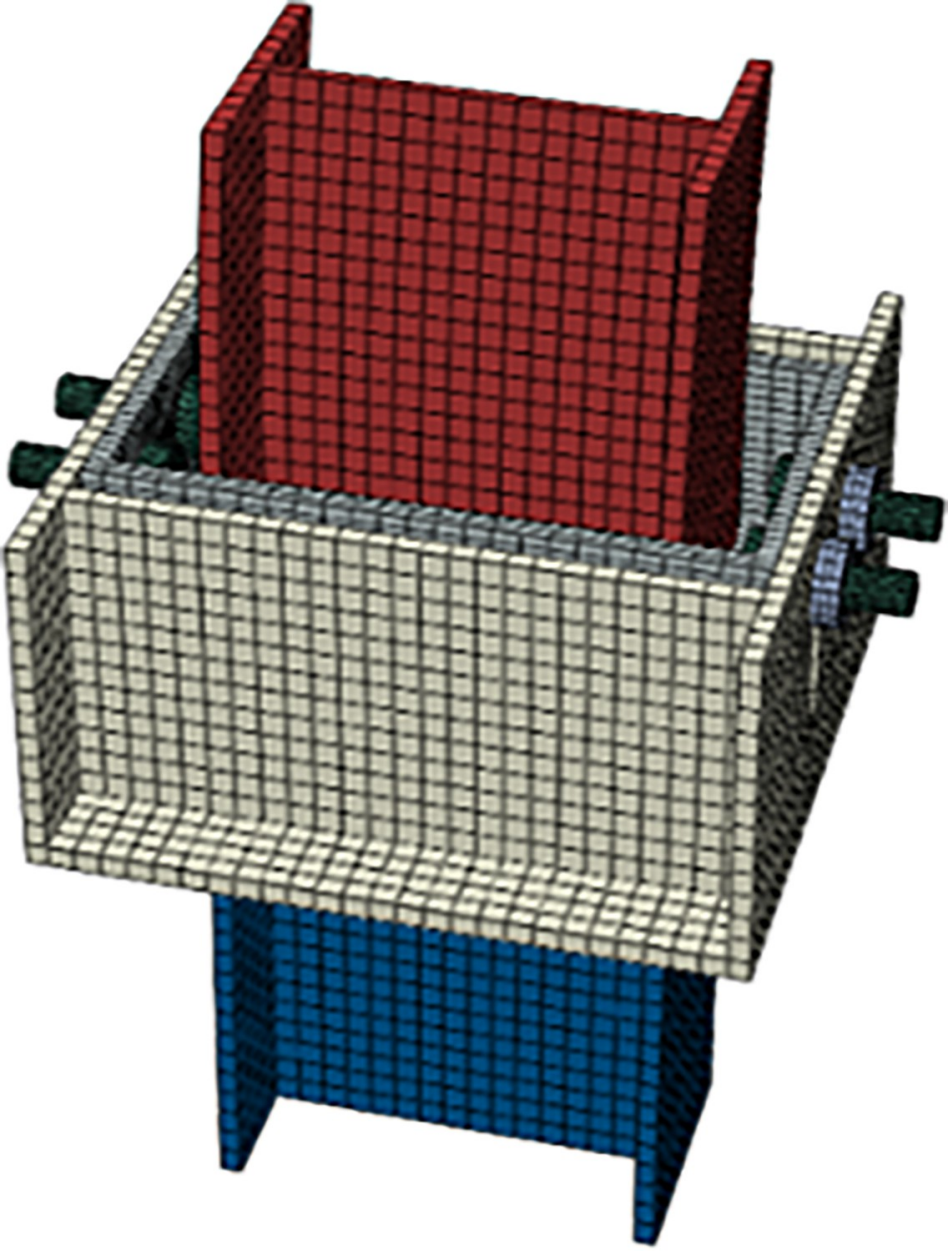
Analysis model of yielding components.

Under the extrusion of surrounding rock, the tunnel support structure is subjected to both radial and axial forces. Dong Biao et al. [23] utilized numerical simulation methods to conduct a detailed analysis of several key performance indicators of the yielding components, including their bending stiffness, compressive strength, and yielding performance. The analysis model of the yielding components is shown in Fig 7.

### 5.1 Radial force effect

**Fig 8:**
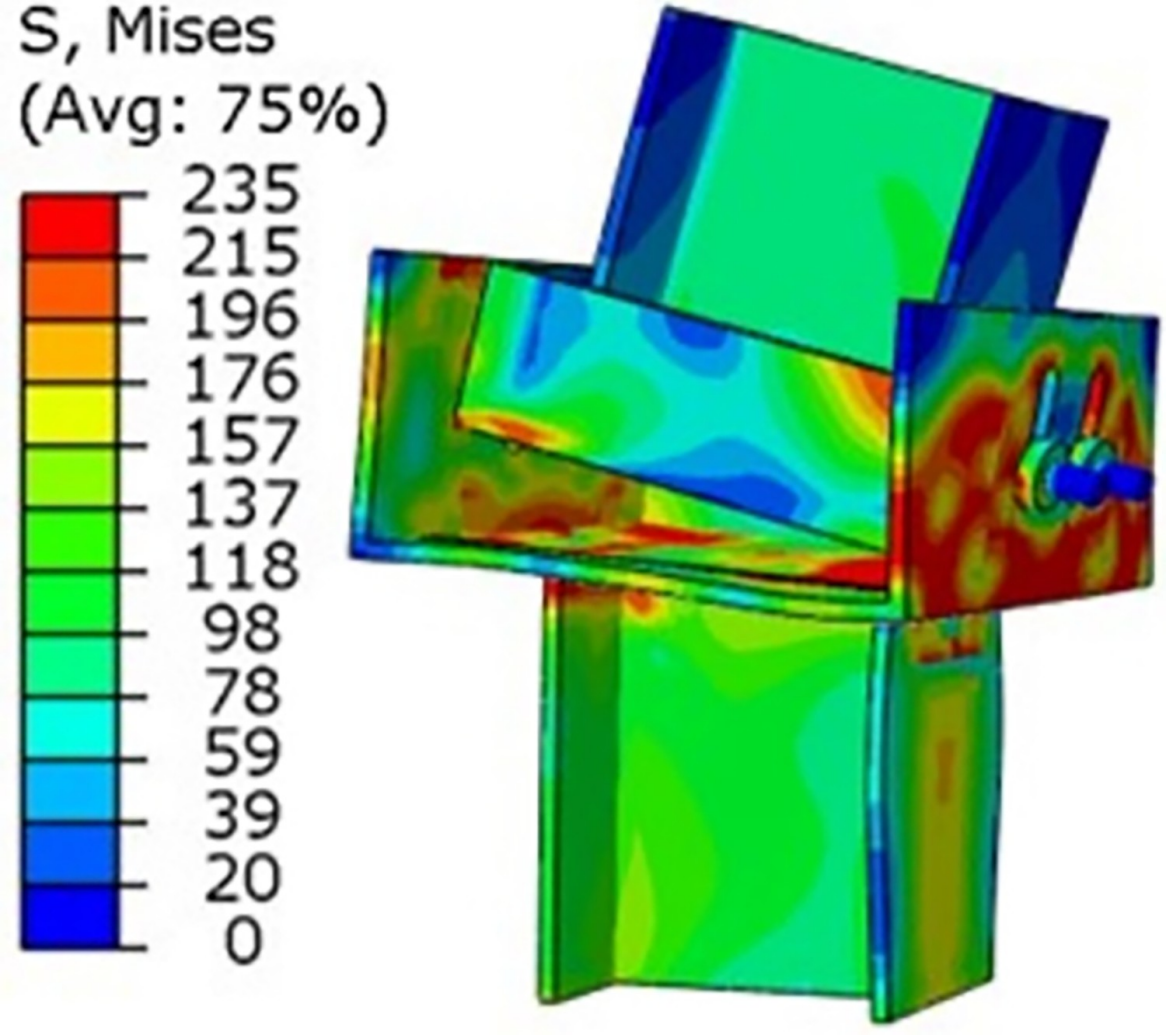
Radial mises stress contour map of yielding components.

A horizontal load of 10kN is applied to the top of the I-beam of the yielding component, and the bolt pre-tension is set to 60KN, resulting in a radial Mises stress contour map of the yielding component (Fig 8). As can be seen from Fig 8, under this load, the yielding component has undergone plastic deformation.

### 5.2 Axial force effect

**Fig 9:**
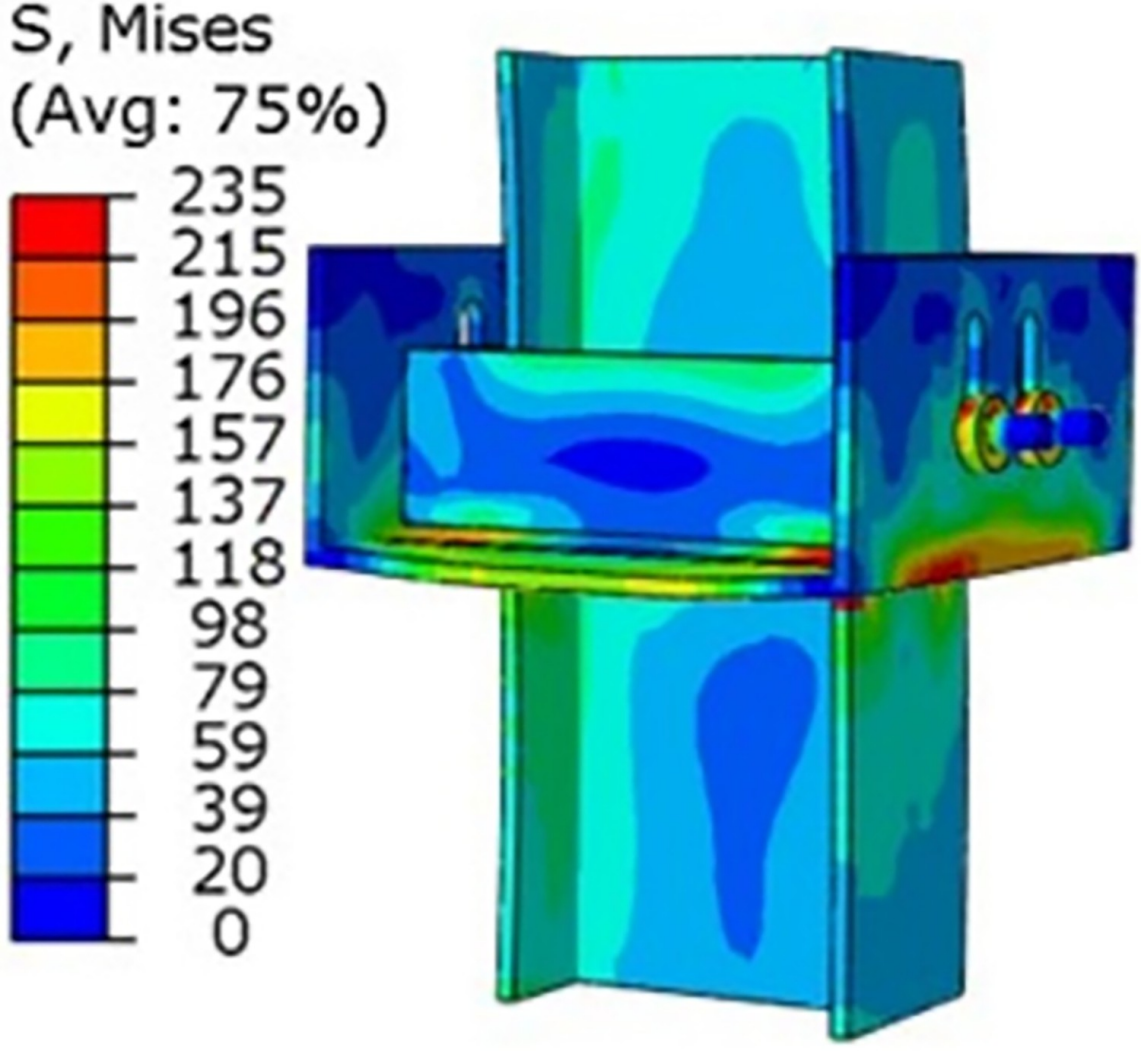
Axial mises stress contour map of yielding components.

A vertical load of 200KN is applied to the top of the I-beam of the yielding component. The bolt pre-tension is the same as during the radial force effect, resulting in an axial Mises stress contour map of the yielding component (Fig 9). As can be seen from Fig 9, under this load, the slot displacement of the yielding component has been fully released.

**Fig 10:**
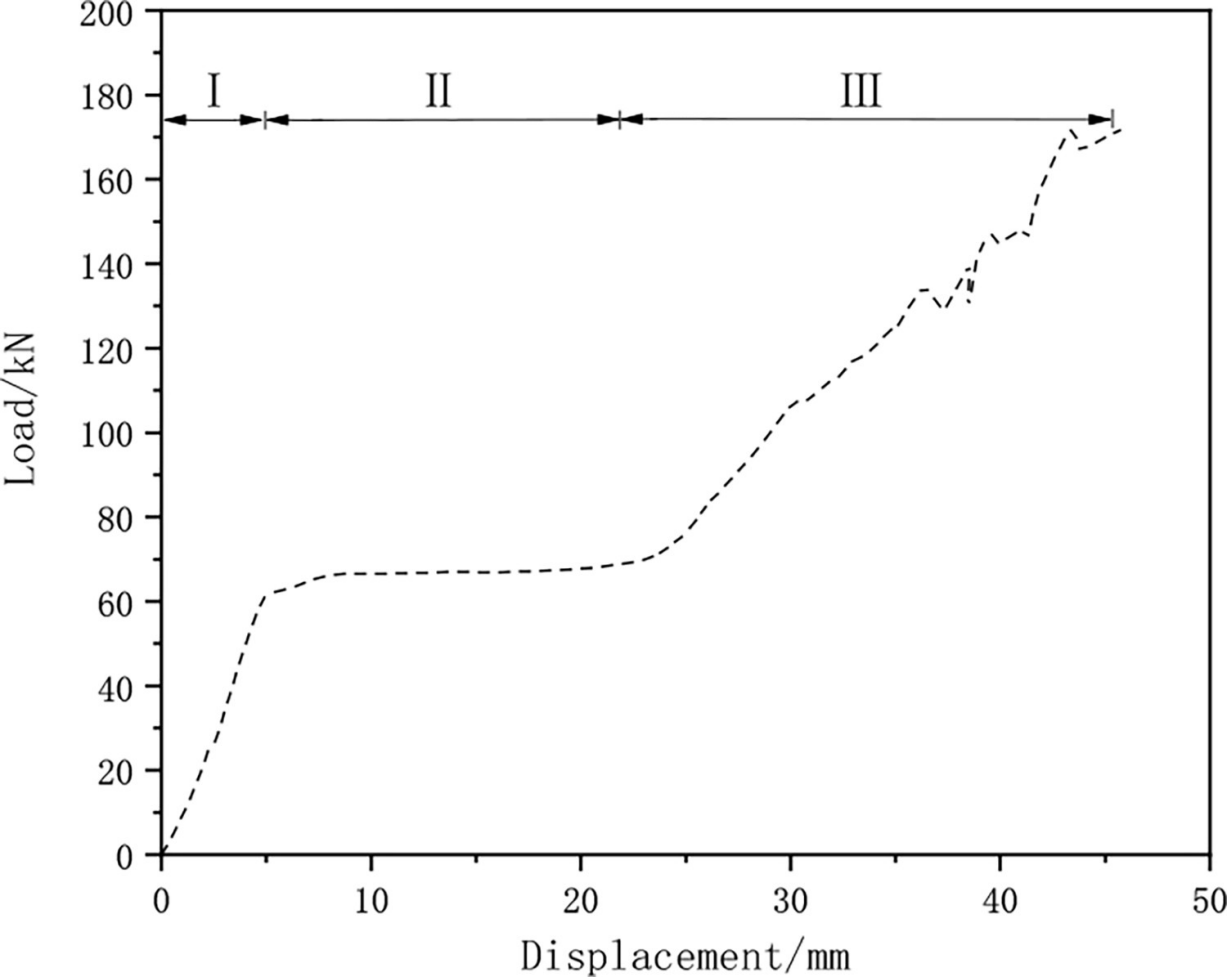
Load-displacement curve of yielding components.

The load-displacement curve of the yielding components is plotted (Fig 10). As shown in Fig 10, the deformation process of the yielding components is divided into three stages: the elastic rise stage of bearing capacity (Ⅰ), during which the bearing capacity gradually increases from 0 to about 60KN with the increase of bolt friction; the constant bearing capacity stage (Ⅱ), where the bearing capacity is maintained within the range of 60kN to 62kN, and the bearing capacity remains stable as the slot smoothly slides; the compaction closing stage (Ⅲ), where the slot displacement of the yielding component has been fully released, the inner and outer slot steels are pressed together and closely combined, and the final bearing capacity stabilizes at 172KN.

The numerical simulation results show that once the yielding component enters the plastic state, the friction between the bolt and the slot steel can provide a constant bearing capacity. When the slot displacement of the yielding component is fully released, the compressed inner and outer slot steels provide a high bearing capacity, and the yielding component can still bear the load.

## 6 Laboratory test of yielding components

### 6.1 Overview of the test

**Fig 11:**
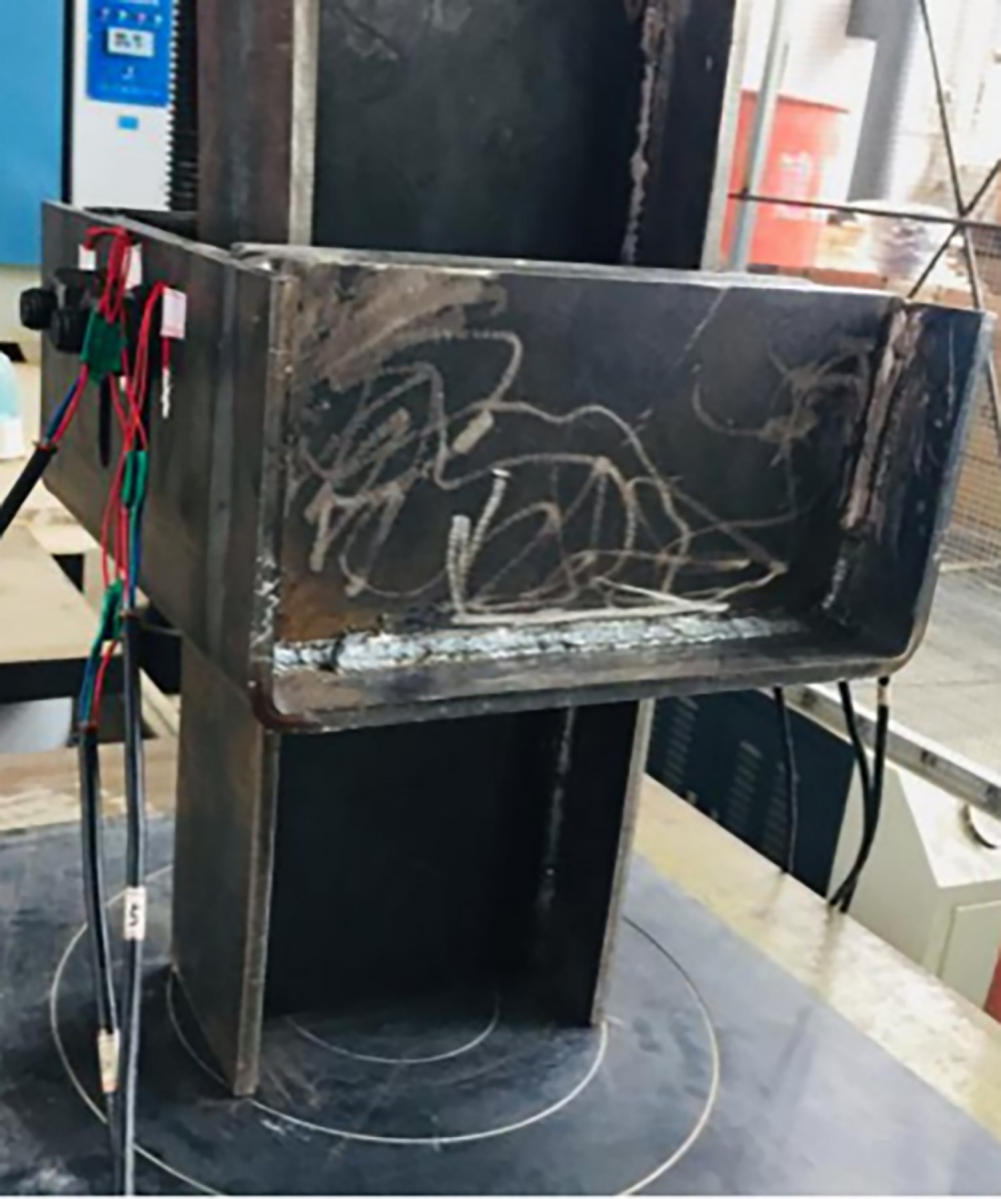
Sample diagram of yielding components.

A microcomputer-controlled electro-hydraulic servo press (YAW-5000F) is used to load the yielding components. The loading method is program-controlled, with a load size of 200 kN and a speed of 1 kN/s. The sample diagram of the yielding component is shown in Fig 11 [23].

### 6.2 Test results

**Fig 12:**
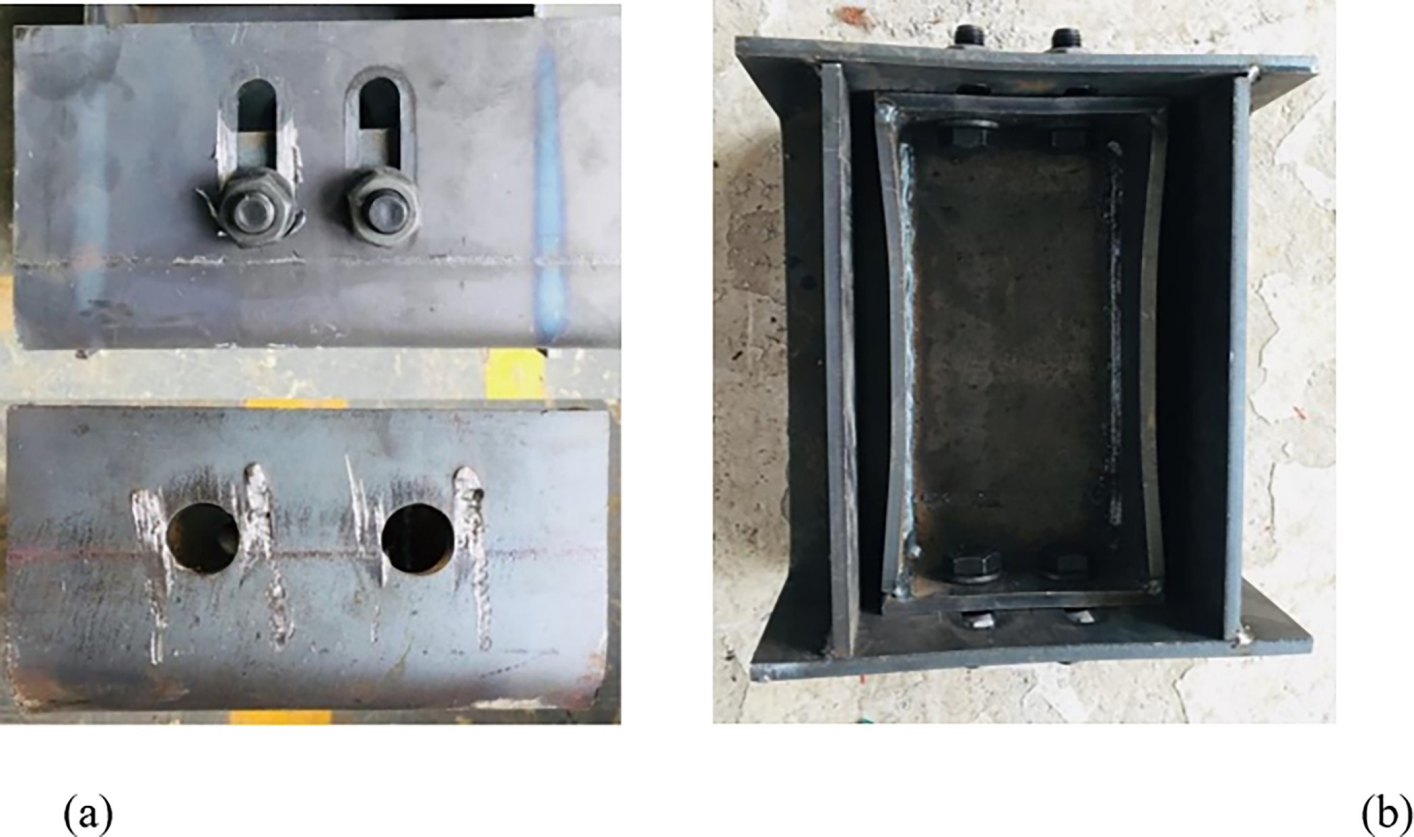
Diagram of the failure form of yielding components. (a) Failure form of slot and bolt hole. (b) Failure form of inner and outer slot steel.

As can be seen from Fig 12, local failure occurred at the slot node, damage occurred on the contact surface between the bolts and the inner and outer slot steels, and the stiffening plate of the inner slot steel bent.After loading, the failure form of the yielding components is shown in Fig 12 [23].

### 6.3 Comparison of results

**Fig 13:**
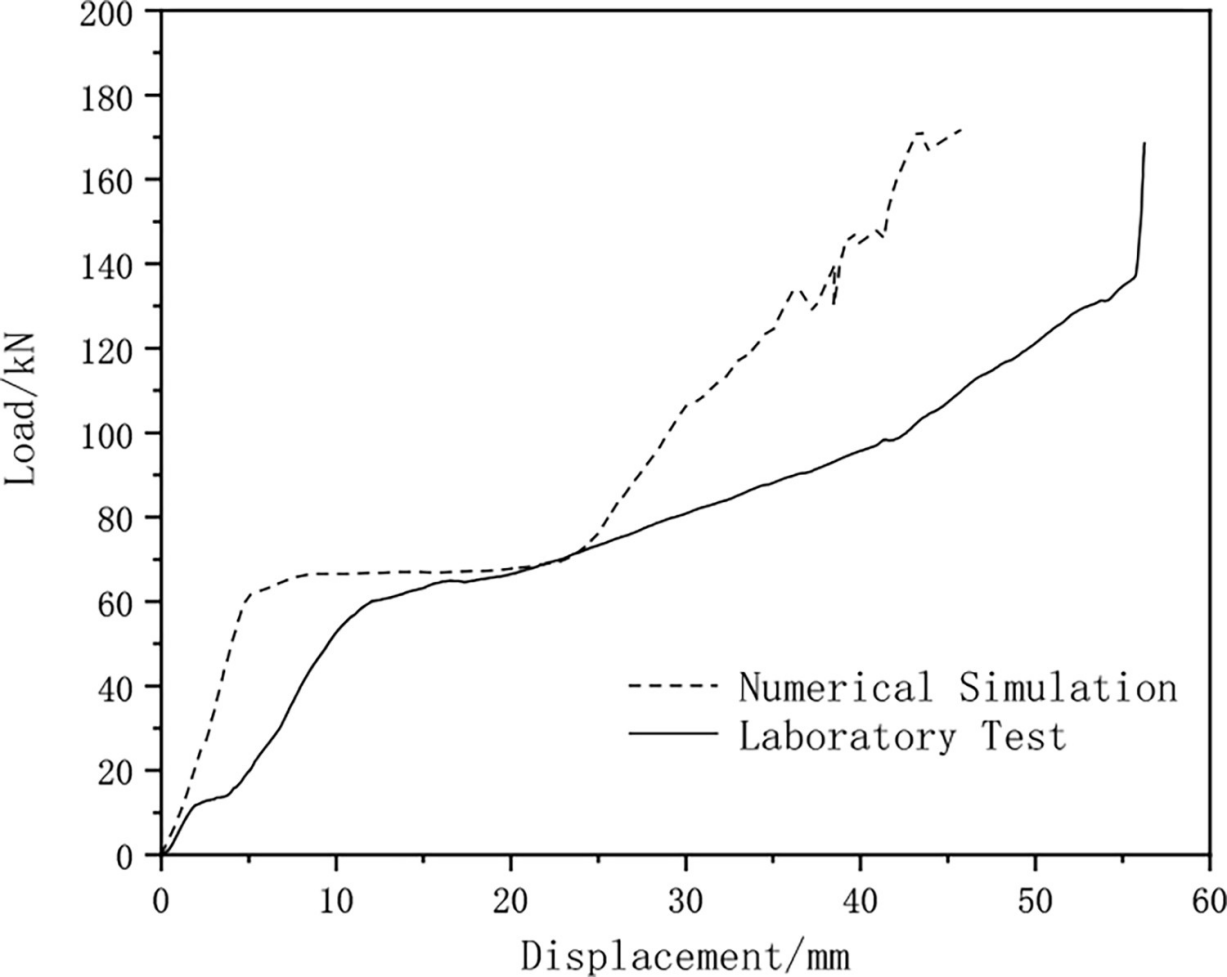
Comparison of displacement-load curves between numerical simulation and laboratory experiments.

The load-displacement curve of the yielding components is plotted and compared with the curve from the numerical simulation results (Fig 13). The comparison shows that the numerical simulation results of the yielding components are highly consistent with the experimental results, but there is a certain numerical difference between them. The main reason for this difference is the manufacturing errors in the slots and bolt holes of the yielding components, such as different roughness of different slot sections.

In the numerical simulation, the deformation of the yielding component when entering the constant bearing capacity stage is 5mm, while in the laboratory test, the deformation is 12mm; however, both stabilize at a load of 60KN when entering the constant bearing capacity stage. After yielding, the numerical simulation and experimental results show maximum load values of 172kN and 169kN, and maximum displacement values of 46mm and 56mm, respectively.

## 7 Case analysis

This paper compares the numerical simulation results of yielding elements with the results of laboratory experiments and uses the interaction models between elastic yielding support and elastic surrounding rock, as well as between elastic yielding support and elasto-plastic surrounding rock, as examples to analyze the entire interaction process between the surrounding rock and yielding support. The model parameters are shown in Table 2.

**Table 2:** Model parameters.

Tunnel Parameters			Yielding Component Parameters
Excavation Radius (*R*/*m*)	Initial Ground Stress (*P*_0_/*Pa*)	Horizontal Stress Coefficient (*λ*)	Area of Connection with Support (*A*/*m*2)
3	1E5	0.8	0.25×10^−3^
Surrounding Rock Parameters			
Elastic Modulus (*E*′/*MPa*)	Poisson’s Ratio (*μ*′)	Cohesion (*C*/*Pa*)	Internal Friction Angle (*θ*)
3	0.25	30000	36°
Support Parameters			
Elastic Modulus (*E*/*MPa*)	Poisson’s Ratio (*μ*)	Support Radius (*r*/*m*)	Position Angle (*φ*)
30000	0.25	2.5	30°

Using the mechanical model shown in Fig 2 as the research object, pressure components are installed at *π*/3, 2*π*/3, 4*π*/3, 5*π*/3, around the ring. The relative displacement at the contact surface between the surrounding rock and pressure-relief support and the support force of the pressure-relief support when reaching a stable state after installing the pressure components are calculated using the displacement approximation method and the support force approximation method. The displacement-load curve of the compression component laboratory test is shown as the ’Laboratory Test’ curve in Fig 13.

### 7.1 Results of the displacement approximation method

**Fig 14:**
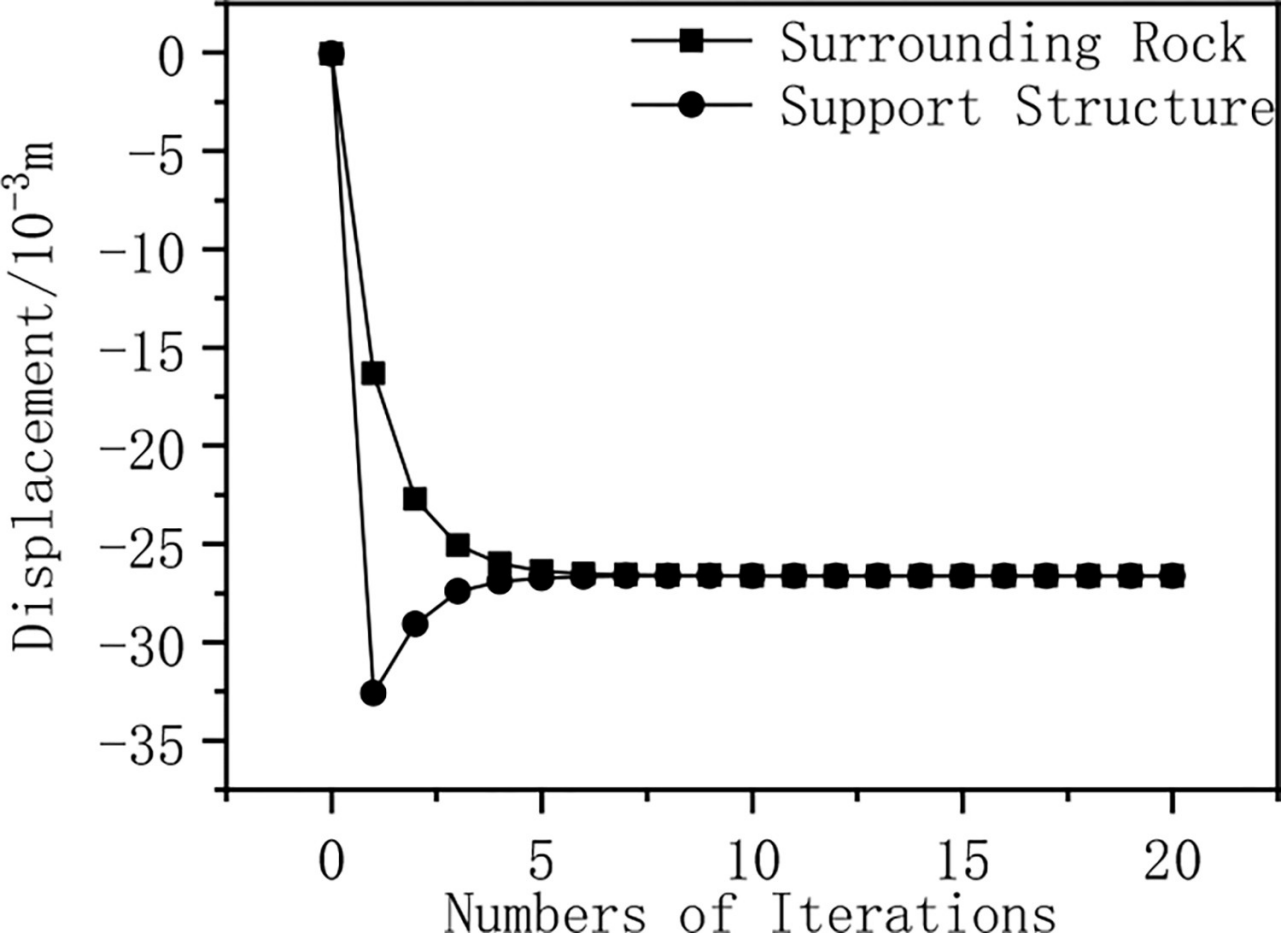
Displacement of elastic surrounding rock and support.

**Fig 15:**
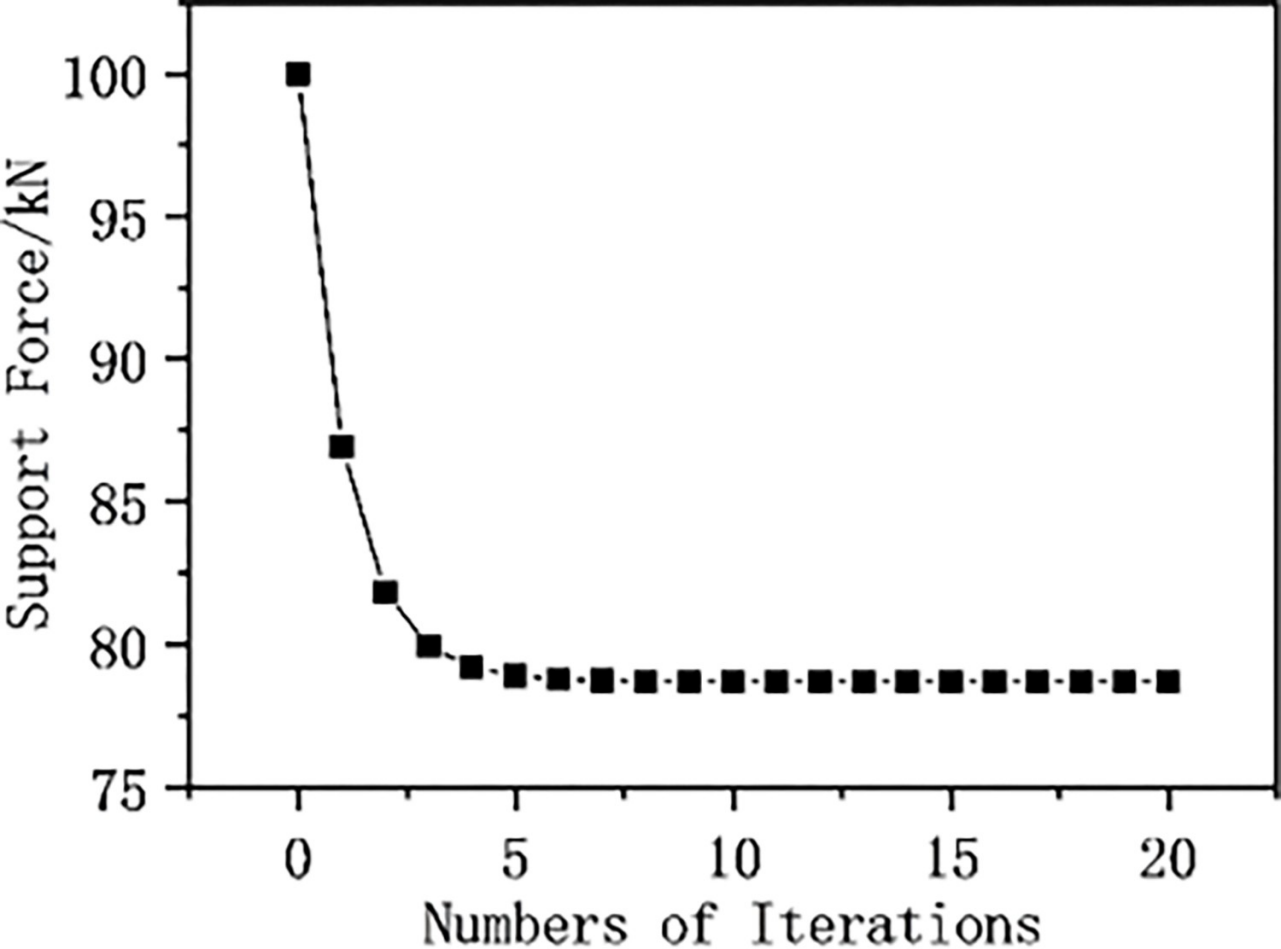
Support force curve of elastic surrounding rock.

The relative displacement values of elastic surrounding rock-rigid support interaction in a stable state are taken as the initial values for iterative calculation (0th iteration). Subsequently, based on the displacement coordination condition and static equilibrium condition, the entire process of the interaction between the surrounding rock and pressure-relief support is iteratively solved. The displacement and support force of the elastic surrounding rock interaction model are calculated using the displacement approximation method, and the relative displacement graph of elastic surrounding rock and support (Fig 14), and the support force curve of elastic surrounding rock (Fig 15) are drawn. As shown in Fig 14, the relative displacement value at the beginning of the iteration is small, and the relative displacement between the surrounding rock and the support increases with the number of iterations; after 5 iterations, the relative displacement between the rock and the support tends to converge. As shown in Fig 15, the initial support force value is 100KN, which decreases with the number of iterations; after 5 iterations, the support force value tends to converge. The calculation results show that after using pressure components in rigid support, the surrounding rock and support transition from a rigid support stable state to a pressure-relief support stable state.

### 7.2 Results of the support force approximation method

**Fig 16:**
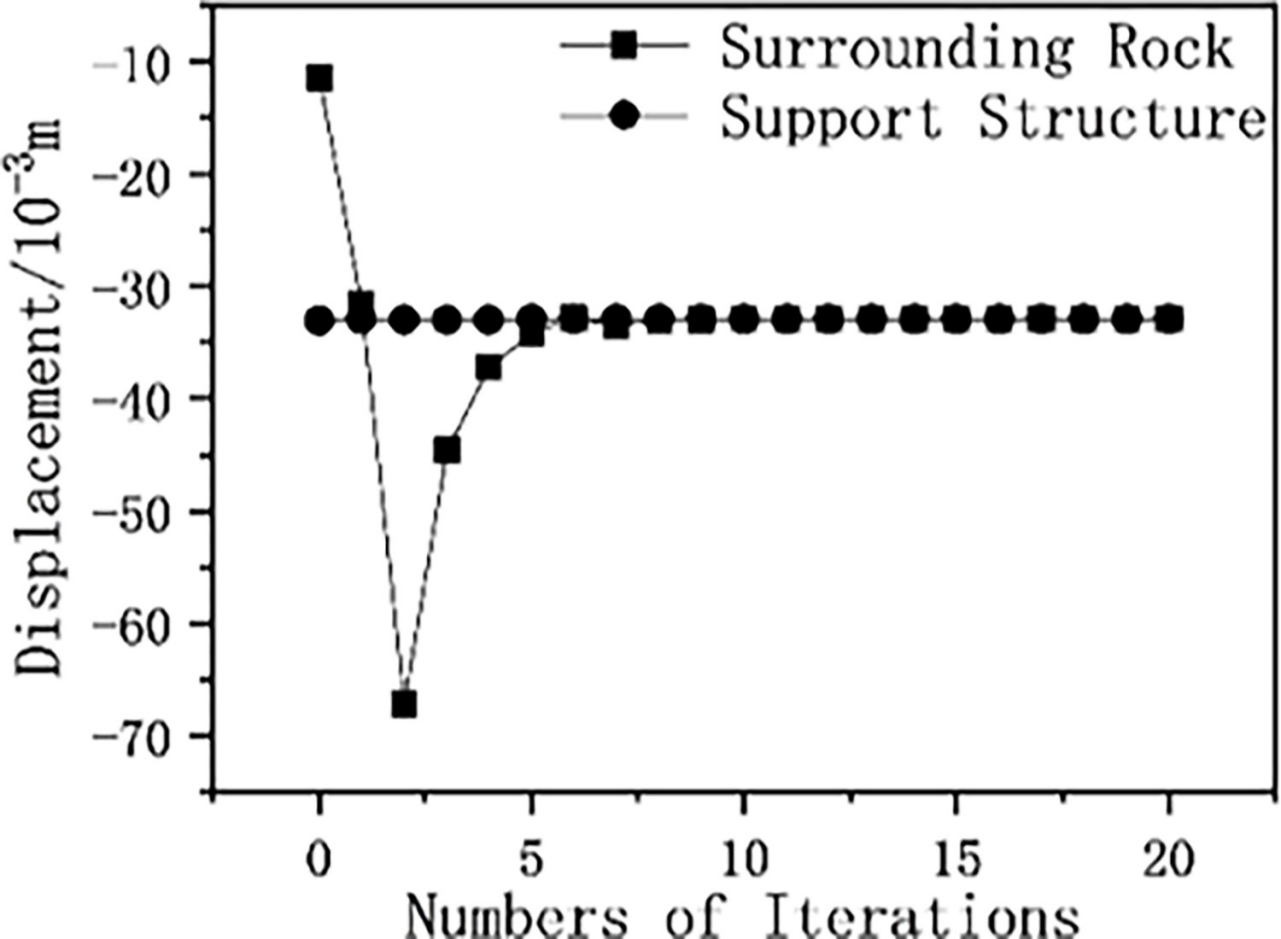
Displacement of elasto-plastic surrounding rock.

**Fig 17:**
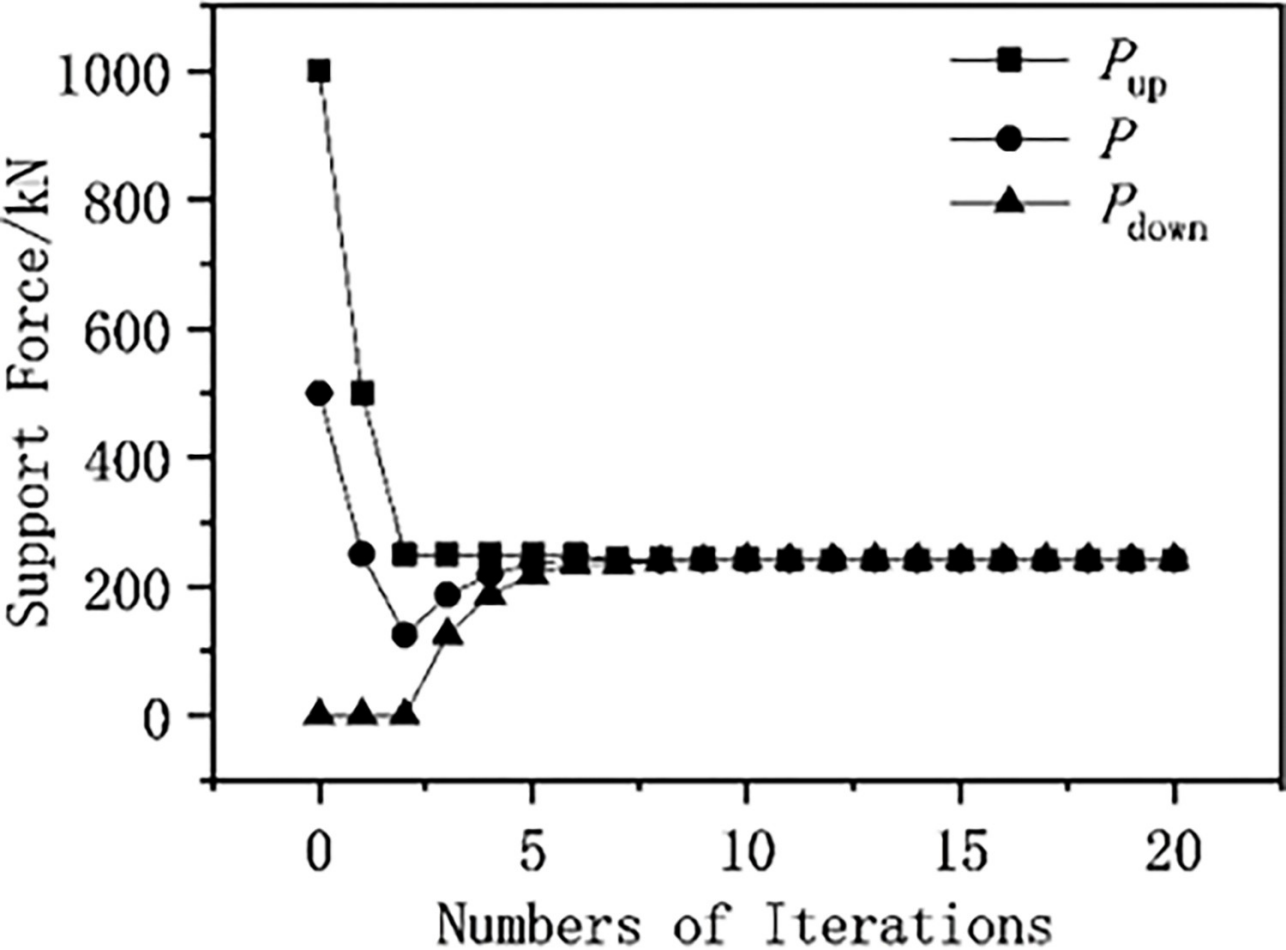
Supporting force and its range.

The lower and upper limits of the support force value are denoted as *P*_down_ and *P*_up_, respectively, with the lower limit being 0 and the upper limit being 10 times the initial geostress. The relative displacement and support force of the elastic-plastic surrounding rock and pressure-relief support interaction model are calculated using the support force approximation method, and the displacement graph of elastic-plastic surrounding rock and support (Fig 16) and the support force and its range change graph (Fig 17) are drawn. As shown in Fig 16, at the 10th iteration, the relative displacement of the surrounding rock and support is equal (meeting the displacement coordination condition). As shown in Fig 17, the range of the support force value is halved with each iteration, and from the 5th iteration, the change in the support force value decreases, converging within no more than 10 iterations.

### 7.3 Convergence of different numerical approximation methods

**Fig 18:**
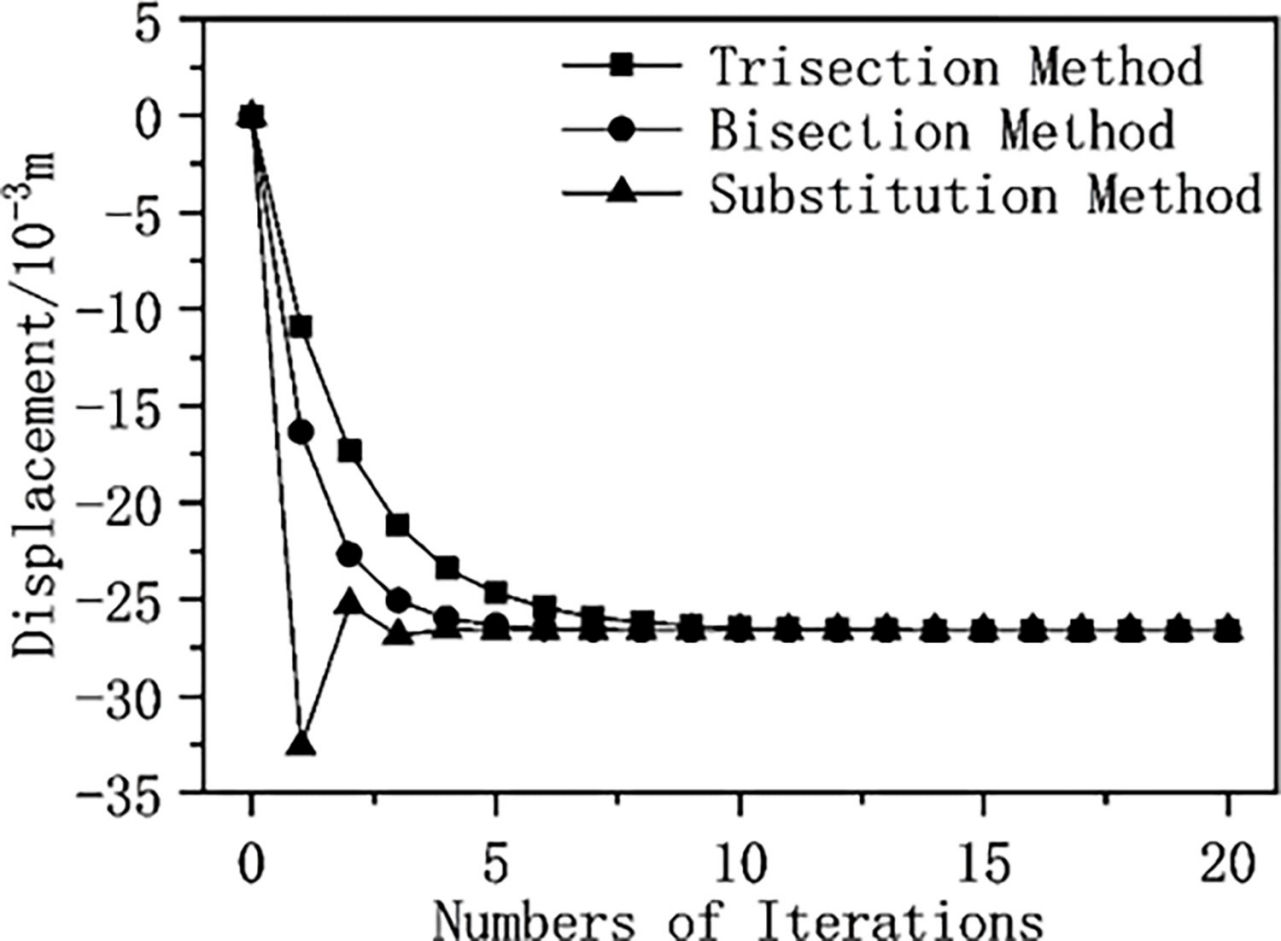
Displacement of different approximation algorithms.

The relative displacement of elastic surrounding rock and pressure-relief support interaction is calculated using the trisection method ($u_{R}^{\prime }=(2u_{R}^{\prime }+u_{R}^{*})/3$), bisection method ($u_{R}^{\prime }=(u_{R}^{\prime }+u_{R}^{*})/2$), and substitution method ($u_{R}^{\prime }=u_{R}^{*}$), and the impact of these three algorithms on the convergence of the calculation results is analyzed by drawing a graph of different approximation methods’ displacement and iteration numbers (Fig 18). From Fig 18, it can be seen that the trisection method has the slowest convergence speed, the displacement curve of the substitution method has sudden changes, and the bisection method has faster convergence speed and higher stability.

## 8 Results and discussion

### 8.1 Stability analysis

The stability of two tunnel cases was analyzed by solving with a numerical approximation algorithm, verifying the accuracy and reliability of the numerical approximation algorithm in calculating the relative displacement and support force at the contact surface between the surrounding rock and yielding support. The results of the numerical approximation algorithm for the two tunnel cases are shown in Table 3.

**Table 3:** Displacement of surrounding rock and support in tunnel cases—numerical approximation algorithm solution results.

	Elastic surrounding rock and support Unit: 10^-3^m		Elasto-plastic surrounding rock and support Unit: 10^-3^m	
Iteration number	Surrounding rock displacement	Support displacement	Surrounding rock displacement	Support displacement
0	-0.05	-0.05	-11.4	-33.07
1	-16.33	-32.61	-31.5	-32.94
2	-22.71	-29.08	-67.23	-33.01
3	-25.06	-27.41	-44.57	-32.97
4	-25.99	-26.92	-37.22	-32.96
5	-26.37	-26.74	-34.18	-32.95
6	-26.52	-26.67	-32.8	-32.96
7	-26.57	-26.62	-33.48	-32.95
8	-26.59	-26.62	-33.14	-32.95
9	-26.61	-26.62	-32.97	-32.95
10	-26.61	-26.62	-32.89	-32.95
11	-26.62	-26.62	-32.93	-32.95
12	-26.62	-26.62	-32.95	-32.95
13	-26.62	-26.62	-32.94	-32.95
14	-26.62	-26.62	-32.94	-32.95
15	-26.62	-26.62	-32.94	-32.95
16	-26.62	-26.62	-32.94	-32.95
17	-26.62	-26.62	-32.94	-32.95
18	-26.62	-26.62	-32.94	-32.95
19	-26.62	-26.62	-32.94	-32.95
20	-26.62	-26.62	-32.94	-32.95

Table 1 shows that the initial displacement changes of the elastic surrounding rock with support are minor, then gradually increase but the growth rate slows down. Compared to the elasto-plastic type, the stability of elastic surrounding rock and support may be achieved more quickly. The initial displacement changes of elasto-plastic surrounding rock with support are larger and then gradually stabilize. As the number of iterations increases, the displacement changes of the surrounding rock and support gradually decrease, tending towards a fixed value.

### 8.2 Comparison of methods

The method in this paper shows significant advantages over traditional methods such as linear superposition in dealing with the interaction between surrounding rock and support structures in tunnels under high ground stress conditions with soft surrounding rock. The displacement approximation method and support force approximation method proposed in this paper better handle the complex issues of displacement coordination and static equilibrium. Although these methods are relatively complex in the calculation process, they provide higher accuracy, which means that in practical applications, it can reduce the repeated calculations and design modifications caused by inaccurate models, thereby saving time and resources.

### 8.3 Existing limitations and challenges

This research also reveals several limitations when proposing new calculation methods: First, the complexity and uncertainty of actual geological conditions may become the main challenges in implementing these methods. Due to the variability of geological conditions, the required models must be flexible enough to adapt to different engineering environments. Second, although these methods perform well in research, their universality in different geological conditions and engineering environments needs further verification, especially in some extreme or uncommon geological conditions, where these methods may need to be adjusted to better fit the actual situation, beyond the basic assumptions of the current model. In addition, the effectiveness of these methods may vary in different practical application scenarios.

Despite these challenges, the methods in this paper provide a computational approach for calculating the interaction between surrounding rock and support structures. Although practical applications may encounter challenges of geological complexity and technical requirements, in the long run, these methods still have room for development in increasing the accuracy of engineering design, ensuring structural safety, and improving economic efficiency.

## 9 Conclusion

To calculate the relative displacement at the contact surface between surrounding rock and pressure-relief support and the support force of pressure-relief support, this paper starts from the interaction mechanism between surrounding rock and rigid support. Based on the displacement coordination condition and static equilibrium condition, the displacement approximation method and support force approximation method are presented. Through the analysis of case studies, the effectiveness of the methods proposed in this paper is verified, and the main conclusions are as follows:

(1) The displacement approximation method and support force approximation method can efficiently and accurately calculate the relative displacement at the contact surface between the surrounding rock and yielding support and the support force of the yielding support. The calculation results not only meet the static equilibrium condition but also the displacement coordination condition, proving the accuracy and reliability of these two approximation methods. In addition, these two methods have fewer iterations and faster convergence.

(2) The convergence of the bisection method, the trisection method, and the substitution method, three numerical approximation algorithms, is comparatively analyzed. The bisection method performs better in terms of stability and convergence speed when solving the interaction problem between surrounding rock and yielding support, showing superiority over the trisection method and substitution method in terms of convergence speed and stability.

(3) This paper adopts a method combining theoretical analysis, numerical approximation algorithms, and laboratory experimental data sets, providing an important reference for the design of yielding support structures in tunnels with soft surrounding rock under high ground stress conditions.

## Supporting information

S1 File (TXT)

S1 Dataset (XLSX)
